# Application of a double-colour upconversion nanofluorescent probe for targeted imaging of mantle cell lymphoma

**DOI:** 10.18632/oncotarget.23860

**Published:** 2017-12-23

**Authors:** Guang Yang, Yong Cao, Bin Yan, Qiang Lv, Jianbo Yu, Fusheng Zhao, Zhihong Chen, Heran Yang, Mengxi Chen, Zaishun Jin

**Affiliations:** ^1^ Pathology Department, Hongqi Hospital of Mudanjiang Medical University, Mudanjiang 157011, P.R. China; ^2^ Key Laboratory of Tumor Prevention and Treatment (Heilongjiang Higher Education Institutions), Mudanjiang Medical University, Mudanjiang 157011, P.R. China

**Keywords:** upconversion fluorescence, nanoprobe, biological imaging, mantle cell lymphoma, immune labelling

## Abstract

Upconversion nanoparticles are a new type of fluorescent marker in biomedical imaging that can convert a longer wavelength (such as near-infrared fluorescence) into a shorter wavelength (such as visible light). Mantle cell lymphoma, which is derived from B-cell lymphoma, is a subtype of non-Hodgkin's lymphoma, and the immune phenotype is a mature B-cell phenotype (CD20+, CD5+). To develop the use of nanomaterials as specific markers for the medical imaging of mantle cell lymphoma, we modified the surface of UCNPs by oxidation so that the CD20 or CD5 antibody could covalently attach to the upconversion nanoparticles to form antibody-UCNP conjugates. These antibody-UCNP conjugates were used as fluorescent probes to detect the CD20 or CD5 antigen. Due to the excessive expression of these antigens on the surface of MCL cells and successful strong connection between the antibody and UCNPs, the latter could specifically combine with mantle cell lymphoma cells. Upon near-infrared excitation at 980 nm, cells labelled with UCNPs emitted bright upconversion fluorescence.

## INTRODUCTION

Mantle cell lymphoma (MCL) is a class of non-Hodgkin's lymphoma (NHL), which shows characteristic changes in pathology, immune phenotype and cell and molecular genetics; it accounts for 6% of all NHLs [1]. t (11; 14) (q13; q32) chromosome translocation is the most unique molecular biological characteristic of MCL, resulting in fusion between the immunoglobulin heavy chain gene on chromosome 14 and the CCND I gene on chromosome 11 and overexpression of the characteristic CyclinD1 in MCL cells [2]. In addition, MCL, which is derived from B-cell lymphoma, is a subtype of NHL; the immune phenotype is a mature B-cell phenotype, and excessive CD20 and CD5 antigens are expressed on the surface of MCL cells [3, 4]. With recent developments in molecular biology and genetics, as well as the clinical use of molecular targeted drugs, the median survival time of MCL patients has increased to 7 years [5]. However, MCL is an aggressive and severely malignant mature B-cell neoplasm, which, in most cases, ends with tumour resistance and relapse [6]. Due to the poor efficacy of conventional treatment, early pathological changes, and rapid invasion, most patients have advanced-stage disease (Ann Arbor stage III–IV) when first diagnosed. Therefore, early detection, early diagnosis and early treatment of MCL are crucial in prolonging the survival time.

Immunolabelling and fluorescence imaging have been widely used in cell biology research and clinical applications and are powerful tools for detailed studies of the interactions among cells and the cellular state [7]. Conventional biomarkers, including organic dyes and fluorescent proteins, have been used in cell and tissue imaging. However, the use of these traditional biomarkers in cell imaging studies is disappointing because these markers experience a high rate of photobleaching [8]. In addition, organic dyes are susceptible to the effects of chemical reactions and metabolism. Thus, their use in cell tracer experiments is greatly restricted [9]. Quantum dots (QDs) have successfully overcome the above shortcomings, but their inherent toxicity and chemical instability have aroused widespread concern [10]. Research [11–13] has shown that conventional biomarkers (organic dyes, fluorescent proteins and QDs) require excitation by an ultraviolet (UV) or a short wavelength light source, leading to a series of shortcomings: (1) the image signal-to-noise ratio (SNR) is very low due to autofluorescence of biological samples; (2) the depth of the excitation light penetration is very shallow; and (3) long-term exposure may cause serious damage to the cells, and even cell death. Molecular imaging technology has been developed for biological structure and functional imaging [14], and by using a two- photon or multiphoton mechanism [15], upconversion nanoparticles (UCNPs) can convert light with longer wavelengths (such as near-infrared light) into light with shorter wavelengths (such as visible light) and can thus be applied in biomedical imaging as fluorescent markers. UCNPs have many advantages, including high quantum yield, narrow emission, powerful upconversion, high detection sensitivity [16–18], good chemical stability, deep tissue penetration depth, no light damage [19, 20] and very low cytotoxicity [21]. Due to the absence of autofluorescence [22], both the SNR and detection sensitivity are obviously increased [23, 24]. Numerous previous *in vitro* cell targeting imaging and *in vivo* animal imaging experiments have confirmed that UCNPs as biological probes can act as a contrast agents with superior performance [25–28]. According to their advantages in terms of basic medical research and clinical applications, UCNPs have the potential to replace traditional fluorescent probes as biological molecular markers in cell targeting imaging and *in vivo* animal imaging [29, 30]. Recently, several researchers have reported on the application of upconversion nanoprobes for biological imaging *in vivo* and *in vitro* [31–33]. In these biological imaging studies, cervical cancer cells, ovarian cancer cells, colorectal cancer cells and KB cells were selected as target cells and good experimental results were achieved. It was confirmed that the fluorescent probe based on an upconversion nanomaterial can be used as a tumour-targeting biomarker in biological imaging applications. However, there is no relevant report on MCL cells and other suspension cells. To the best of our knowledge, this is the first report on the use of UCNPs for MCL medical imaging. It was previously reported [3, 4] that CD20 and CD5 antigens are usually located in the MCL cell membrane; therefore, these antigens can be used as specific targets for nanoparticles. When UCNPs that emit different colours of light were functionalized with CD20 and CD5 antibodies and were used to specifically label CD20 and CD5 antigens on the surface of MCL cells, the accuracy of MCL diagnosis was significantly improved. In the present study, we investigated the application of double-colour UCNPs in cell-targeted imaging *in vitro* and animal imaging *in vivo*.

## RESULTS

### Characterization of UCNPs

**Figure 1:**
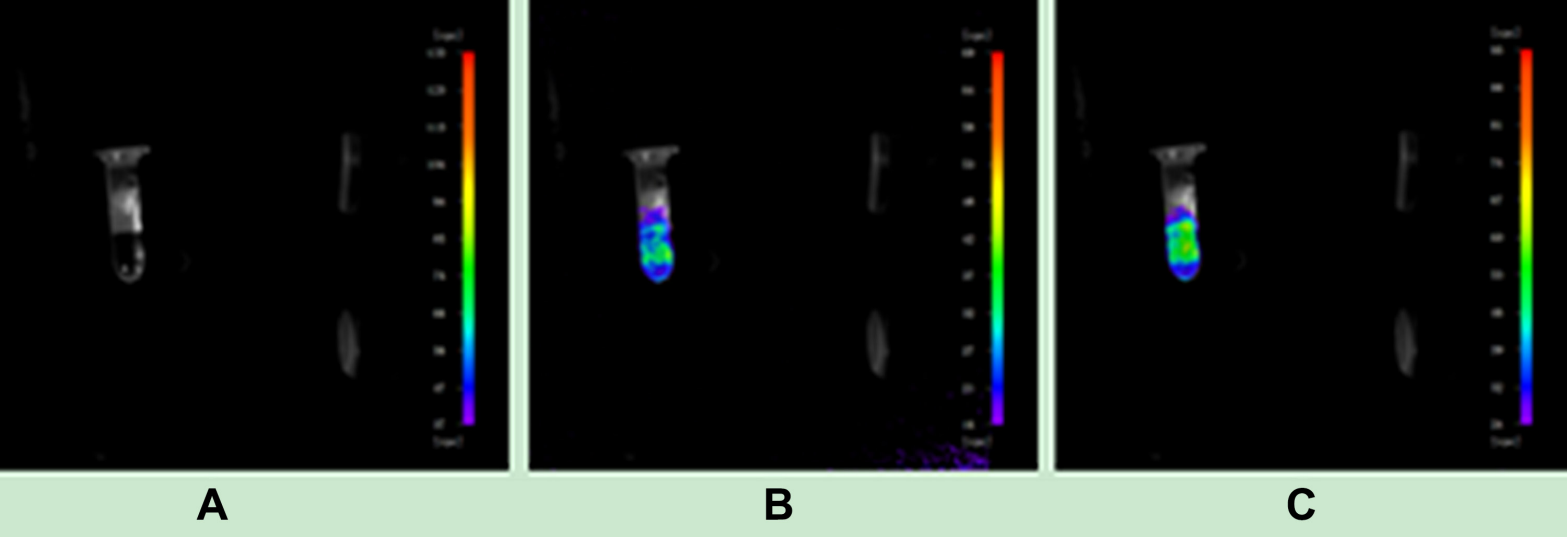
Upconversion imaging of UCNPs (**A**) Imaging with no near-infrared laser excitation. (**B**) Imaging with a near-infrared laser excitation power of 0.7 W. (**C**) Imaging with a near-infrared laser excitation power of 1 W.

We extracted a small amount of the UCNP suspension coupled with the corresponding antibody to determine the upconversion performance of the nanoparticles using a NightOWL small-animal *in vivo* imaging system with 980 nm near-infrared (NIR) excitation. As shown in Figure 1, the UCNPs did not produce upconversion fluorescence (UCL) without NIR excitation. When the excitation power of NIR light was 0.7 W, the UCNPs released bright UCL. When the excitation power was increased to 1 W, the intensity of UCL was stronger.

**Figure 2:**
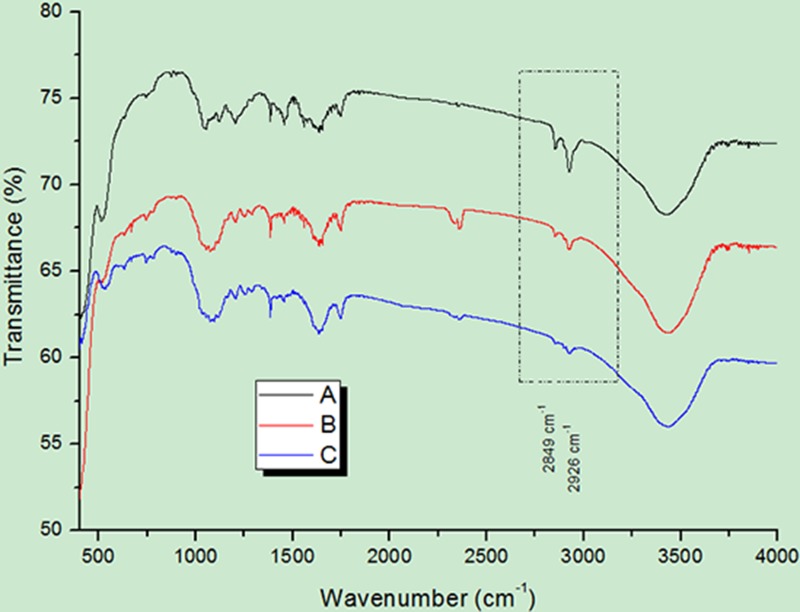
FT-IR spectral analysis of UCNPs (**A**) FT-IR spectrum of oxidized UCNPs by H2O2. (**B**) FT-IR spectrum of UCNPs reacted with NHS and EDC. (**C**) FT-IR spectrum of UCNPs reacted with antibody.

In addition, characterization of the nanoparticles was also performed using Fourier transform infrared (FT-IR) spectrometry, which is an indirect detection method [26], to verify whether the UCNPs had been modified and covalently coupled with the corresponding antibody successfully. As shown in Figure 2A–2B and 2C represent the spectra of oxidized UCNPs alone, UCNPs reacted with NHS and EDC, and UCNPs reacted with antibody, respectively. At 2849 cm^−1^ and 2926 cm^−1^, the intensities of the absorption peaks of the three different treated particles gradually decreased from A to C.

**Figure 3:**
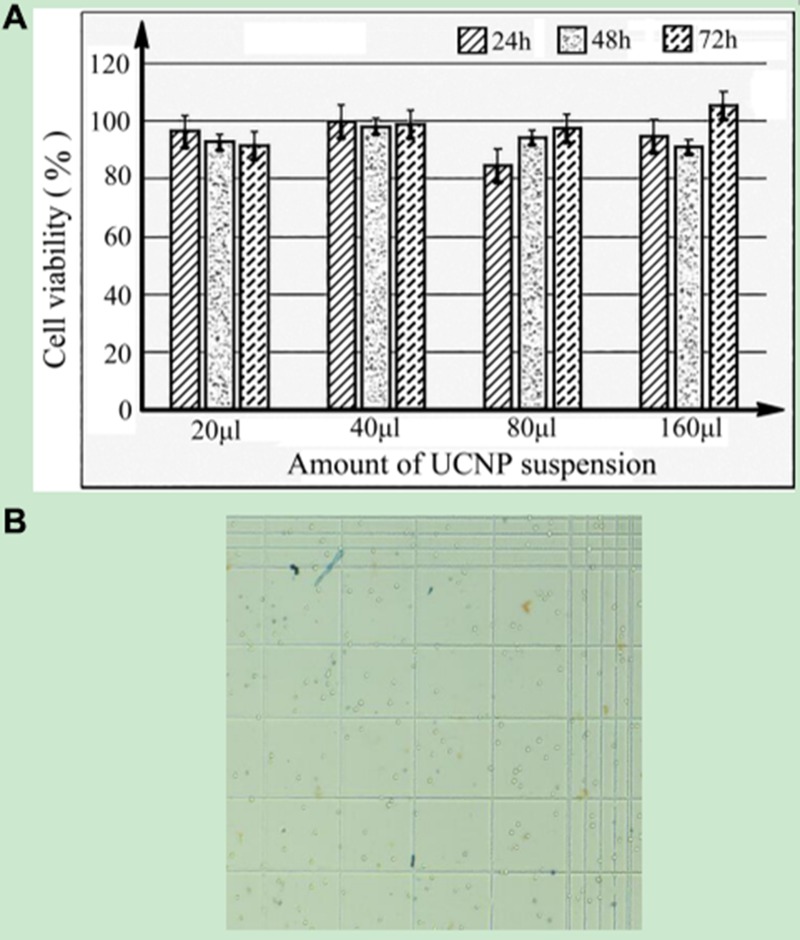
(**A**) Viability of Jeko-1 cells when they were cultured with different amounts of UCNPs suspension at 37°C and 5% CO_2_ for 24 h, 48 h and 72 h. (**B**) Imaging of viable cells when Jeko-1 cells were cultured with 160 μl of UCNP suspension for 72 h.

To detect the cytotoxicity of UCNPs, we used Trypan blue to stain dead cells to evaluate cell growth after co-culture of UCNP-CD20 antibody conjugates and cells at different time periods. The amount of UCNP suspension was increased from 20 μl to 160 μl, and then the UCNPs were cultured with the cells for 24 h, 48 h and 72 h. The cells were counted, and cell viability was calculated using a formula mentioned in MATERIALS AND METHODS 3.3. As shown in Figure 3A, cell viability did not significantly decrease with increasing amounts of the UCNP suspension or as the culture time was prolonged. The cell viabilities were almost all above 90%. Figure 3B shows that few dead cells were stained with Trypan blue on the blood cell counting plate, while the number of bright and viable cells was significantly higher.

### Cell immunolabelling and imaging

**Figure 4:**
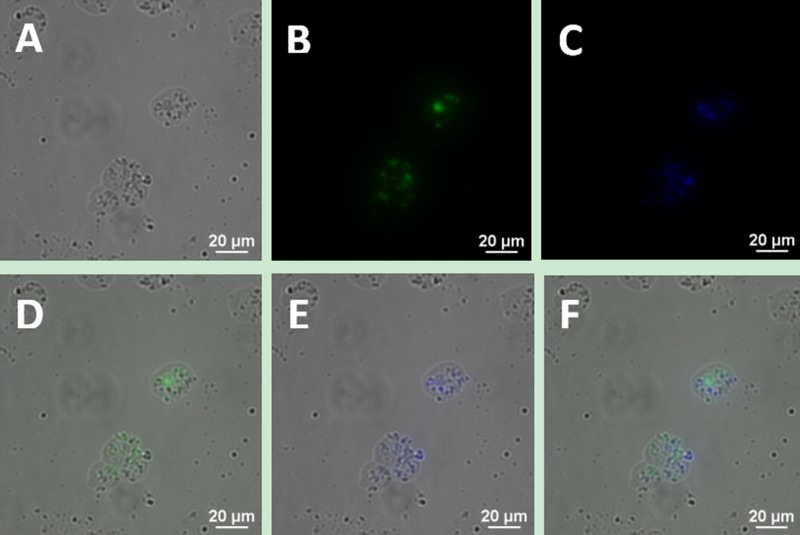
Upconversion fluorescence images of Jeko-1 cells after incubation of cells with a mixture including the UCNP-CD20 antibody conjugates and UCNP-CD5 antibody conjugates for 2 h (**A**) Bright field imaging; (**B**) Dark field fluorescence imaging in the green channel; (**C**) Dark field fluorescence imaging in the blue channel; (**D**) Overlap of A and B; (**E**) Overlap of A and C; (**F**) Overlap of D and E.

**Figure 5:**
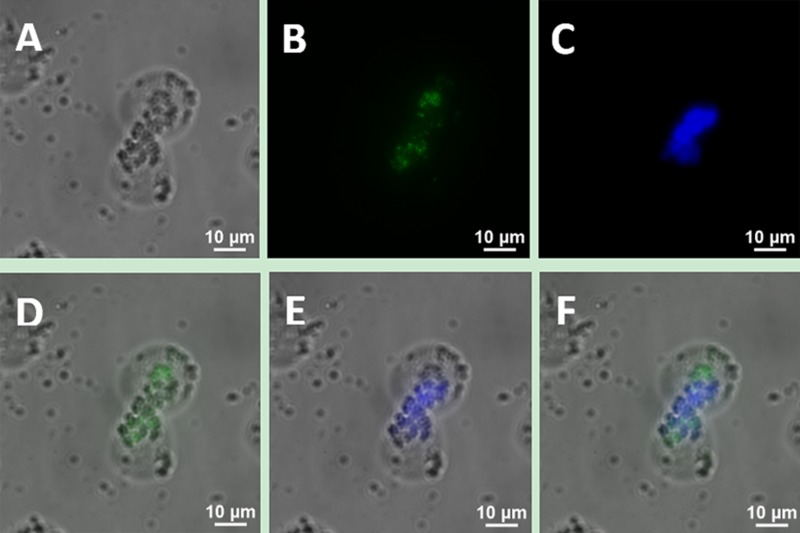
Upconversion fluorescence images of Jeko-1 cells after incubation of the cells with a mixture containing UCNP-CD20 antibody conjugates and UCNP-CD5 antibody conjugates for 2 h (**A**) Bright field imaging; (**B**) Dark field fluorescence imaging in the green channel; (**C**) Dark field fluorescence imaging in the blue channel; (**D**) Overlap of A and B; (**E**) Overlap of the A and C; (**F**) Overlap of D and E.

To determine whether the bio-functionalized UCNPs can be used as immune markers to label the MCL cell line, we incubated Jeko-1 cells with 300 μl of UCNP suspension, including 150 μl of CD5 antibody-NaYF_4_: Yb^3+^/Tm^3+^ nanoprobe conjugate suspension and 150 μl of CD20 antibody-NaYF_4_: Er3+ nanoprobe conjugate suspension, and the cells were co-cultured for 2 h at room temperature. Following incubation, the cells were imaged using a confocal microscope with 980 nm NIR excitation with an excitation power and exposure time of 1.5 W and 2 s, respectively. As shown in Figure 4, the cells emitted bright blue and green dual-colour UCL, and this luminescence did not overlap on the surface of cells under a 20× objective. Additionally, no luminescent nanoparticles were seen in the extracellular area. Under a 40× objective, luminescent nanoparticles on the surface of the cells were clearly visible (Figure 5).

**Figure 6:**
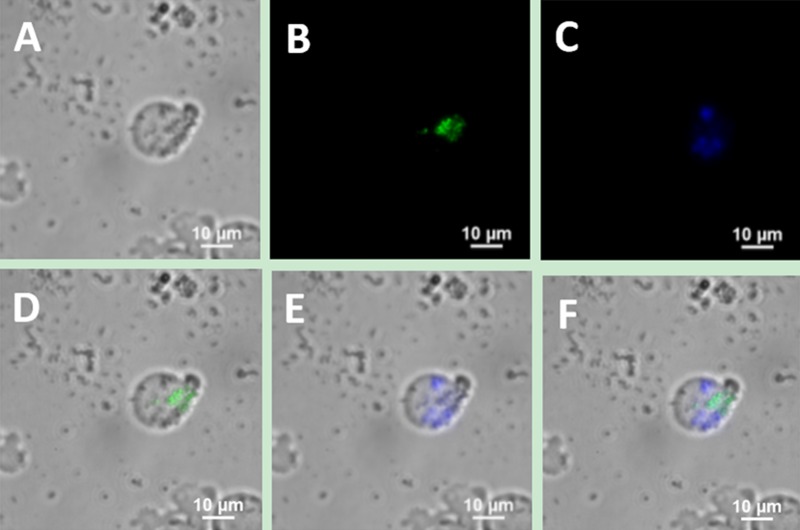
Upconversion fluorescence images of SP50B cells after incubation of the cells with a mixture containing UCNP-CD20 antibody conjugates and UCNP-CD5 antibody conjugates for 2 h (**A**) Bright field imaging; (**B**) Dark field fluorescence imaging in the green channel; (**C**) Dark field fluorescence imaging in the blue channel; (**D**) Overlap of A and B; (**E**) Overlap of A and C; (**F**) Overlap of D and E.

Therefore, we also used another MCL cell line, SP50B [34], to repeat the above experiment. Figure 6 shows that bright blue and green double-colour UCL was observed on the surface of SP50B cells under the 40× objective, which was consistent with the experimental results obtained using Jeko-1 cells.

**Figure 7:**
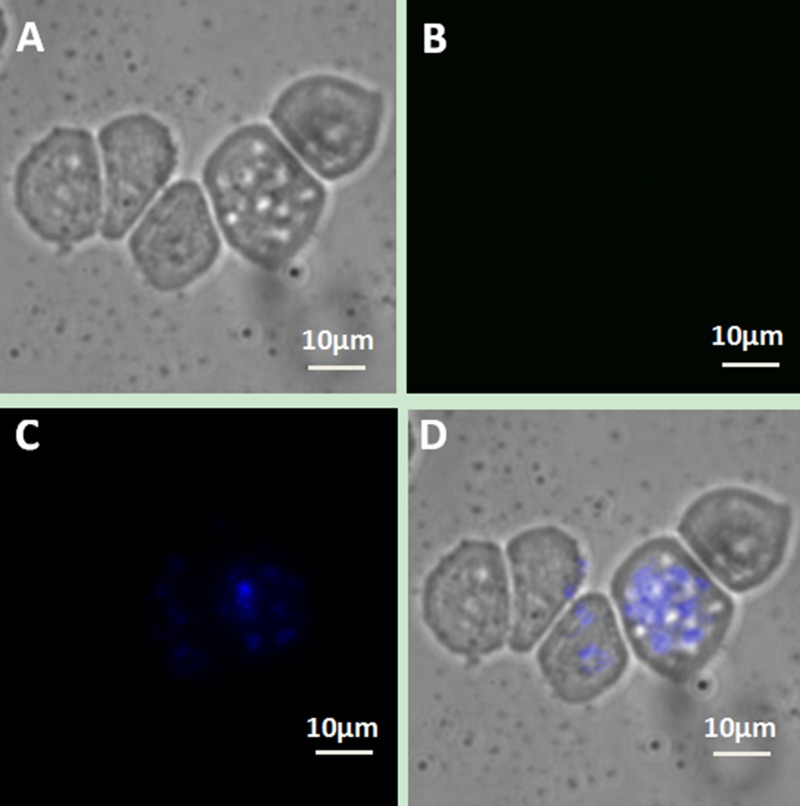
Upconversion fluorescence images of Jeko-1 cells after incubation of the cells with a mixture containing NaYF^4^:Er3^+^ nanoparticle suspension without CD20 antibody and CD5 antibody-NaYF^4^: Yb3^+^/Tm3^+^ nanoparticle suspension for 2 h (**A**) Bright field imaging; (**B**) Dark field fluorescence imaging in the green channel; (**C**) Dark field fluorescence imaging in the blue channel; (**D**) Overlap of A, B and C.

**Figure 8:**
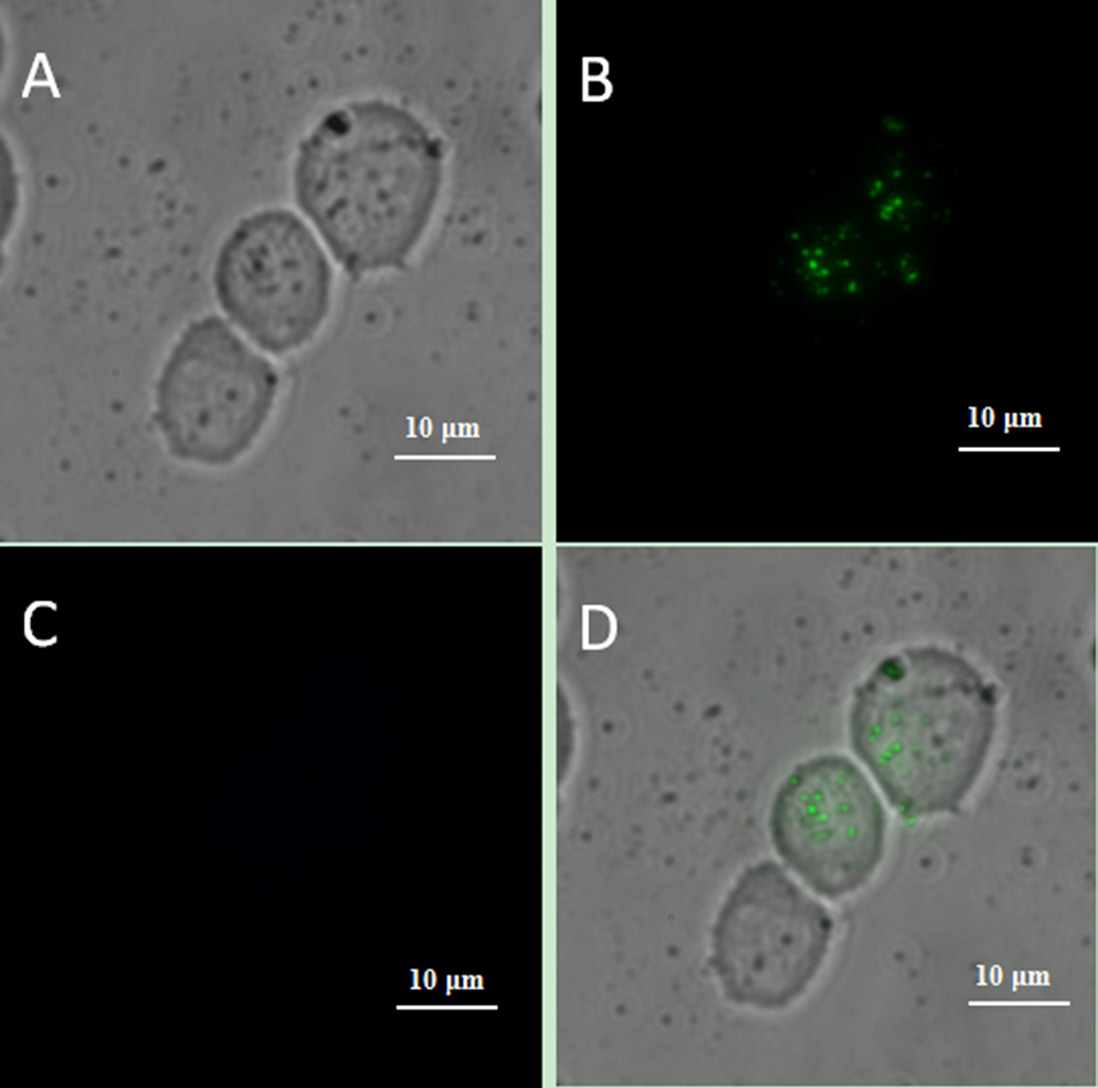
Upconversion fluorescence images of Jeko-1 cells after incubation of the cells with a mixture containing CD20 antibody-NaYF_4_: Er^3+^ nanoparticle suspension and NaYF_4_: Yb^3+^/Tm^3+^ nanoparticle suspension without CD5 antibody for 2 h (**A**) Bright field imaging; (**B**) Dark field fluorescence imaging in the green channel; (**C**) Dark field fluorescence imaging in the blue channel; (**D**) Overlap of A, B and C.

**Figure 9:**
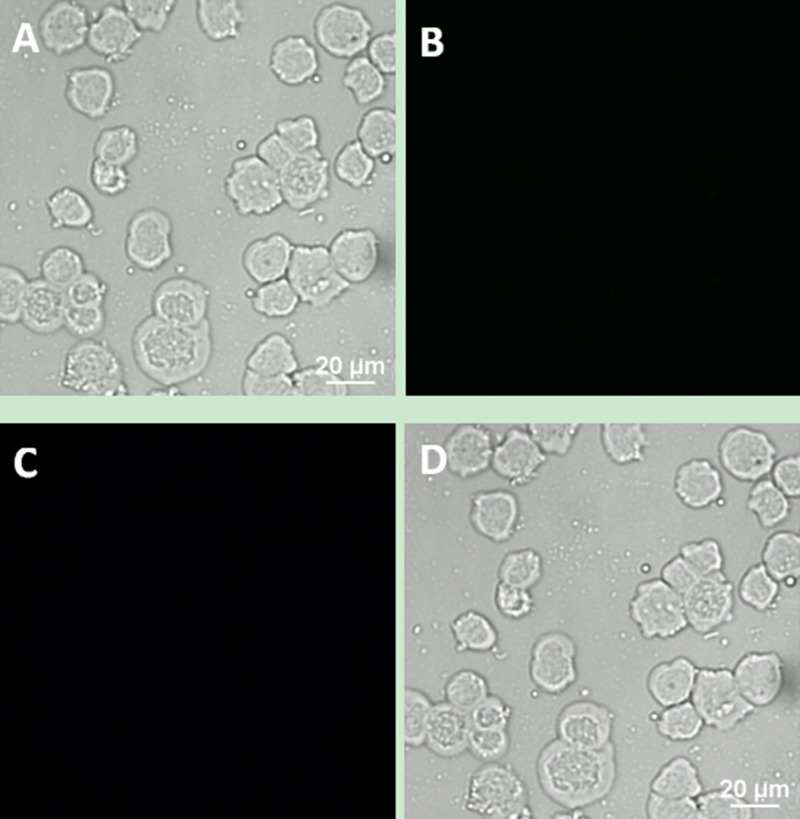
Upconversion fluorescence images of Jeko-1 cells after incubation of the cells with a mixture containing NaYF^4^: Er^3+^ nanoparticle suspension and NaYF_4_: Yb^3+^/Tm^3+^ nanoparticle suspension without any antibody for 2 h (**A**) Bright field imaging; (**B**) Dark field fluorescence imaging in the green channel; (**C**) Dark field fluorescence imaging in the blue channel; (**D**) Overlap of A, B and C.

We designed a series of controlled experiments to confirm that the connection between the cells and UCNPs was based on the specific immune response mediated by antigen and antibody. Using the same incubation conditions, Jeko-1 cells were incubated with a mixture of the NaYF_4_: Er^3+^ nanoparticle suspension without CD20 antibody and the CD5 antibody-NaYF_4_: Yb^3+^/Tm^3+^ nanoparticle suspension. As shown in Figure 7, only bright blue UCL from NaYF_4_: Yb^3+^/Tm^3+^ UCNPs was observed on the cell surface, and no green UCL from NaYF_4_: Er^3+^ was seen on the cell surface. In addition, Jeko-1 cells were incubated with a mixture of the CD20 antibody-NaYF_4_: Er^3+^ nanoparticle suspension and the NaYF_4_: Yb^3+^/Tm^3+^ nanoparticle suspension without CD5 antibody. Fluorescence imaging was performed after a certain period, as shown in Figure 8. Only bright green UCL from NaYF_4_: Er^3+^ UCNPs was observed on the cell surface, and no blue UCL from NaYF_4_: Yb^3+^/Tm^3+^ was seen on the cell surface. Finally, Jeko-1 cells were incubated with a mixture of the NaYF_4_: Er^3+^ nanoparticle suspension and the NaYF_4_: Yb^3+^/Tm^3+^ nanoparticle suspension without antibody. Fluorescence imaging was performed after a certain period, as shown in Figure 9. On the surface of the cells, no UCL colour was observed.

### *In vivo* imaging of mice

**Figure 10:**
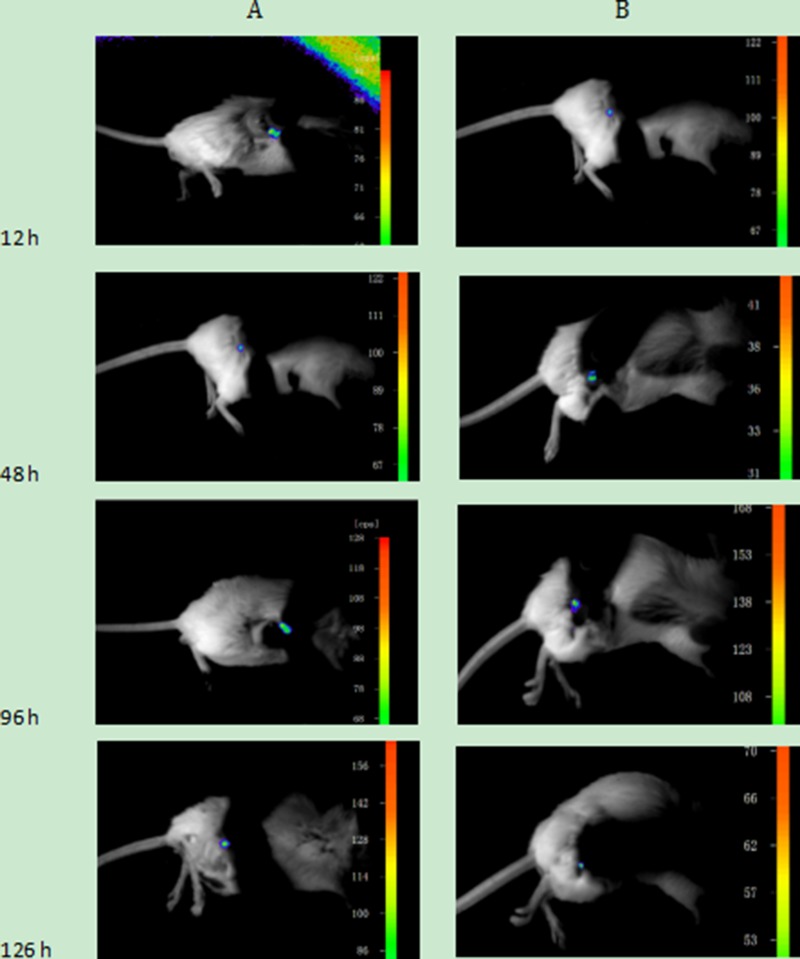
(**A**) Upconversion imaging of a mouse that was subcutaneously inoculated with Jeko-1 cells after tail vein injection of UCNP-CD20 antibody suspension for 12 h, 48 h, 96 h, and 126 h. (**B**) Upconversion imaging of a mouse that was subcutaneously inoculated with Jeko-1 cells after the tail vein injection of the UCNP-CD5 antibody suspension for 12 h, 48 h, 96 h, and 126 h.

**Figure 11:**
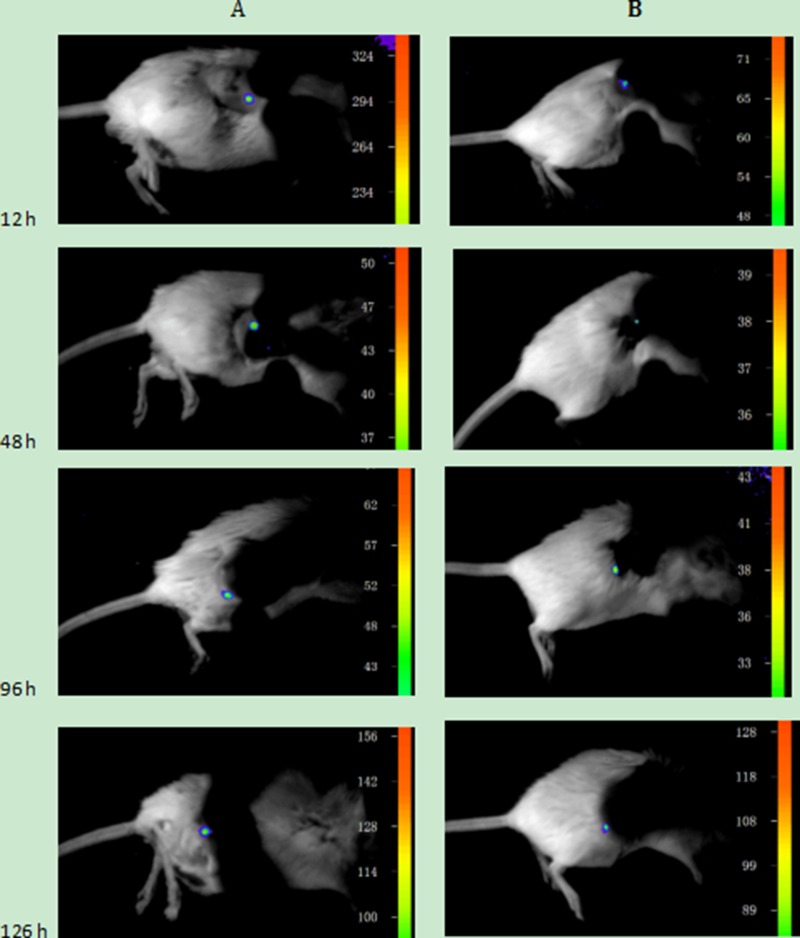
(**A**) Upconversion imaging of a mouse that was subcutaneously inoculated with SP50B cells after the tail vein injection of the UCNP-CD20 antibody suspension for 12 h, 48 h, 96 h, and 126 h. (**B**) Upconversion imaging of a mouse that was subcutaneously inoculated with SP50B cells after tail vein injection of the UCNP-CD5 antibody suspension for 12 h, 48 h, 96 h, and 126 h.

**Figure 12:**
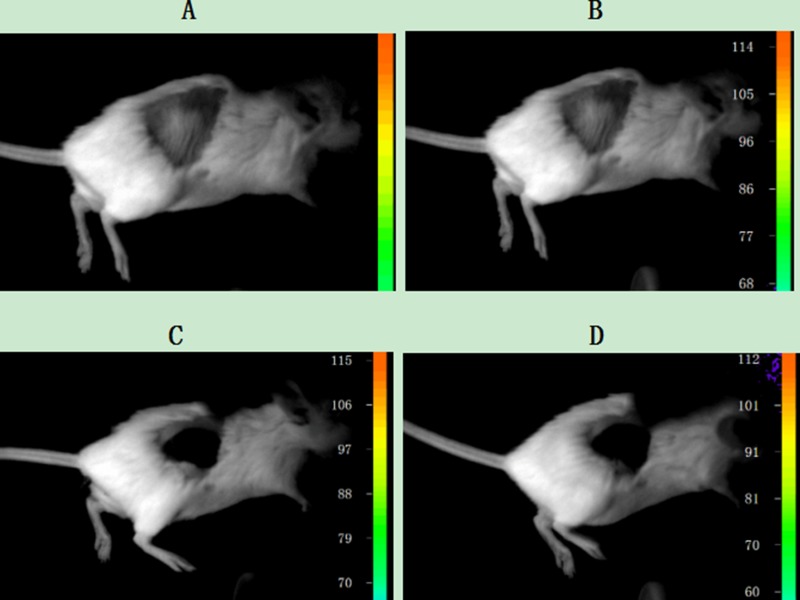
Upconversion imaging of a mouse that was subcutaneously inoculated with SP50B cells after tail vein injection of the UCNP suspension without any antibody (**A**) Imaging without NIR; (**B**) Upconversion imaging with NIR after tail vein injection of the UCNP suspension without any antibody after 48 h; (**C**) Upconversion imaging with NIR after tail vein injection of the UCNP suspension without any antibody after 96 h; (**D**) Upconversion imaging with NIR after tail vein injection of the UCNP suspension without any antibody after 115 h.

**Figure 13:**
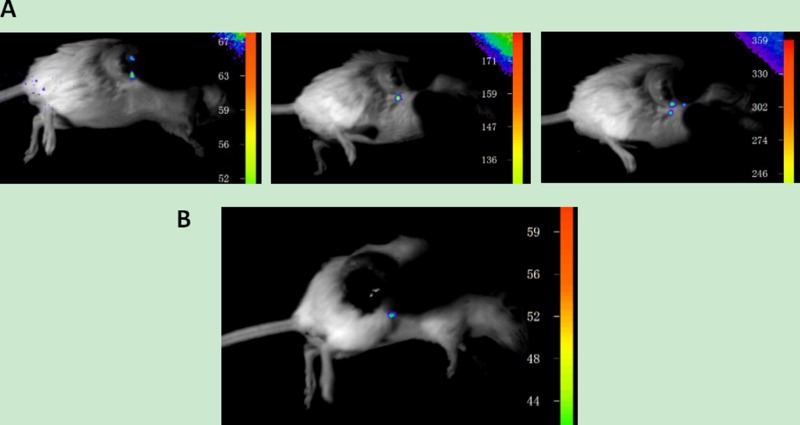
(**A**) Upconversion imaging from the same mouse after changing the laser spot on the mouse tumour. (**B**) Upconversion imaging of a mouse that was subcutaneously inoculated with Jeko-1 cells after tail vein injection of the UCNP-CD5 antibody suspension for 220 h.

Before carrying out the animal experiments *in vivo*, it was confirmed that the connection between the cells and UCNPs was due to the specificity of the immune response. Next, the nanoparticles were further used to detect tumour lesions in mice. Two six-week-old SCID Beige SPF mice were subcutaneously injected with the Jeko-1 cell suspension (cells number: 2×10^7^). After 1 month, the back mass was approximately 0.5 cm in diameter. One mouse was injected with 500 μl of CD20 antibody-NaYF_4_: Er^3+^ nanoparticle suspension through the tail vein, and the other was injected with 500 μl of CD5 antibody-NaYF_4_: Yb^3+^/Tm^3+^ nanoparticle suspension via the tail vein. Following intravenous injection of UCNPs-antibody conjugate suspension, the mice were anaesthetized and imaged *in vivo* 12 h, 48 h, 96 h, and 126 h after injection using the NightOWL small-animal *in vivo* imaging system equipped with a 980-nm NIR laser whose excitation power and exposure time were 3 W and 10 s, respectively. As shown in Figure 10, bright light was seen at the tumour site, and with increasing time up to 220 h, the luminescence intensity at the mass showed no attenuation or quenching (Figure 13B). Similarly, we used the SP50B cell suspension to repeat the above experiments under the same conditions. As shown in Figure 11, we obtained the same results. In addition, we performed control experiments *in vivo*. One mouse that had been subcutaneously injected with the SP50B cell suspension (cell number: 2 × 10^7^) was given the UCNP suspension without antibody via the tail vein, and then upconversion imaging of the tumour at different time points was performed (Figure 12). No bright UCL was observed at the tumour site.

### Imaging of organs after embedding in wax and HE staining of sections

**Figure 14:**
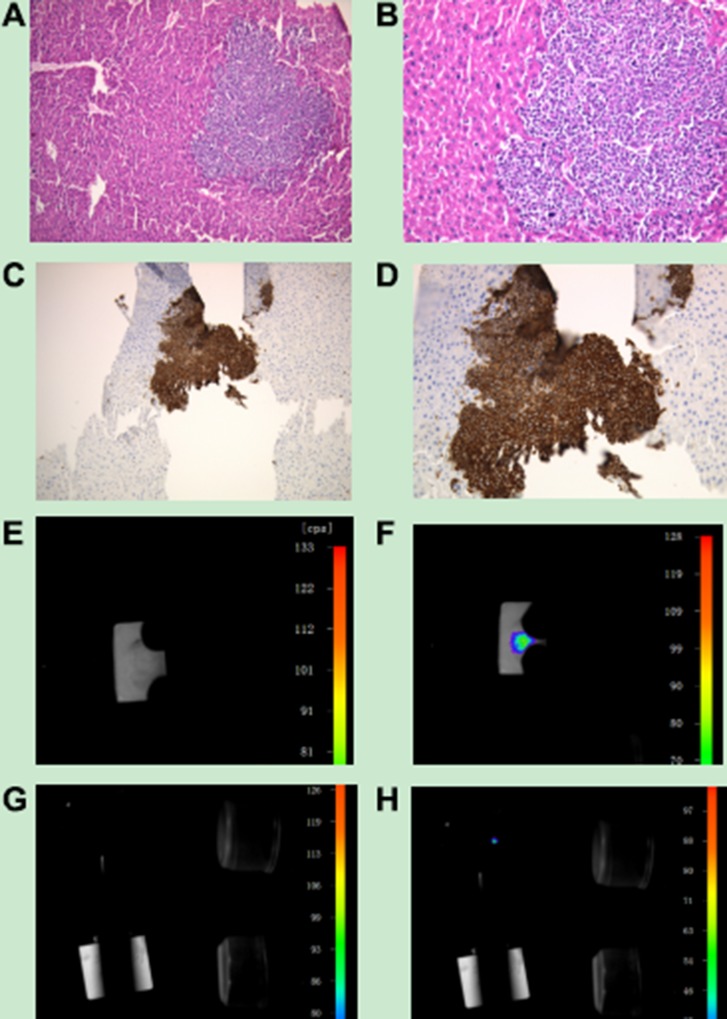
(**A**, **B**) HE imaging of tumour metastasis located in the liver. A: Observation using a 10× objective; B: Observation using a 20× objective. C, D: Immunohistochemistry imaging of tumour metastasis located in the liver. (**C**) Observation using a 10× objective; (**D**) Observation using a 20× objective. (**E**) Paraffin-embedded liver tissue imaging without near-infrared laser excitation; (**F**) Paraffin-embedded liver tissue imaging with near-infrared laser excitation; (**G**) HE slice imaging of liver tissue without near-infrared laser excitation; (**H**) HE slice imaging of liver tissue with near-infrared laser excitation.

We hypothesized that thought UCNP-antibody conjugates could be used for the targeted detection of tumour metastasis in other organs. Three weeks after the administration of the conjugates, the mice were euthanized. The liver, spleen, kidney, heart, brain and lung were extracted, and sections were prepared for HE and immunohistochemistry staining. Microscopy showed that pieces of focuses were not found in other organs but were found in the liver (Figure 14A–14B)). In addition, we performed immunohistochemistry experiments. The focus was stained to be sepia in the liver, futher confirming that the focus was tumour metastasis (Figure 14C–14D). Subsequently, we used the NightOWL small-animal *in vivo* imaging system with 980 nm NIR excitation to perform upconversion imaging of liver tissue embedded in wax and HE slices. As shown in (Figure 14E–14G), without near-infrared laser excitation, the wax and HE slices did not emit UCL. However, with near-infrared laser excitation, the wax blocks and sections showed a strong patchy and spot-like fluorescence, respectively, as shown in (Figure 14F–14H).

**Figure 15:**
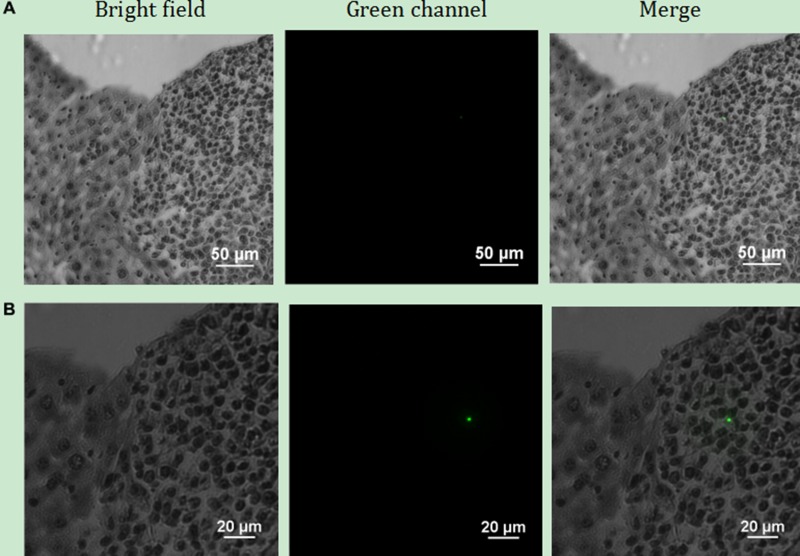
Upconversion imaging of the HE slice of liver tissue using the Ti-S Eclipse inverted fluorescence microscope with a 980 nm near-infrared laser (**A**) Observation using a 10× objective; (**B**) Observation using a 20× objective.

Finally, we used a Ti-S Eclipse inverted fluorescence microscope with a 980 nm NIR excitation to study the position relationship between the nanoparticles and cells in the HE slices. As shown in Figure 15, compared with that of normal cells, the morphology of tumour cells was significantly different. We found that the tumour cells grew densely, and the colour of the nuclei was darker than that of the surrounding normal cells. Observed using a 10× objective, bright UCL was seen among tumour cells, but we did not observe UCL among normal liver cells. When observed with a 20× objective, the UCL of tumour cells was brighter and clearer.

## DISCUSSION

Throughout the experiment, we confirmed that UCNPs have a strong upconversion function. Although the excitation power is very low, these UCNPs can still convert low-energy NIR light into high-energy visible light. Additionally, with an increase in excitation power, the intensity of UCL emitted from UCNPs will also be enhanced. Due to the presence of oleic acid on the surface of the original UCNPs [35], these UCNPs were characterized as hydrophobic. To maintain water solubility, we used hydrogen peroxide to oxidize the carbon-carbon double bond (R-HC = CH-R,) located in the oleic acid ligand on the surface of nanoparticles so that the nanoparticles contained azelaic acid with more carboxyl (-COOH) groups. Thus, the nanoparticles can bind to NHS and EDC to achieve coupling with the antibody. As shown in Figure 3, at 2849 cm^−1^ and 2926 cm^−1^, the intensities of the absorption peaks of three differently treated particles gradually decreased from A to C, indirectly implying that UCNPs were eventually successfully coupled with the antibody. Therefore, the oxidation of hydrogen peroxide on the UCNPs and successful combination of NHS, EDC and UCNPs laid the foundation for the successful coupling between UCNPs and monoclonal antibodies. In addition, we applied a method [36] named Trypan blue cell staining to calculate the rate of cell proliferation and further evaluate the cytotoxicity of UCNPs. From Figure 3, we can see that the cell viability did not decrease obviously when the cells were incubated with UCNPs for a certain period. Therefore, we believe that UCNPs are non-toxic in a certain range and show no inhibitory effect on cell proliferation. This finding was consistent with that of a previous study [21].

In this study, due to a condensation reaction [31] between the amino groups on the surface of nanoparticles and carboxyl groups on the antibody, as well as the presence of NHS and EDC, UCNPs after oxidation were firmly crosslinked to antibodies so that they can specifically combine with an antigen on the surface of cells (Supplementary Figure 1).

When UCNPs coupled with CD5 and CD20 antibodies were incubated with the MCL cell line (Jeko-1, SP50B) after a certain period followed by upconversion imaging, double-colour bright UCL was observed on the cell surfaces (Figures 4, 5, 6). In theory, this suggests that CD5 antibody-NaYF_4_:Yb^3+^/Tm^3+^ UCNPs and CD20 antibody-NaYF_4_: Er^3+^ UCNPs can combine with MCL cells. However, this result did not mean that the connection between UCNPs and cells was a specific combination based on specific immune responses mediated by antigens and antibodies. We conducted three control trials and found that if UCNPs without coupling with a certain antibody were incubated directly with cells, the surface of the cells did not emit the corresponding UCL. Therefore, from cell experiments *in vitro*, we conclude that antibodies play a crucial role in the process of UCNP binding to cells. In other words, the binding of UCNPs to cells is a specific combination based on specific immune responses mediated by antigens and antibodies. Additionally, we also performed animal experiments to verify the above conclusion in mice. Only when UCNPs were coupled with the corresponding antibody did the tumours in the backs of the mice could emit UCL. Thus, we conclude that antibody-functionalized UCNPs can be used as probes to specifically identify target cells and lesions. Over time, UCL showd no attenuation, proving that UCNP-antibody conjugates did not result in light bleaching [37], as shown in (Figure 13B).

Because the animal imaging system only has two laser-transmitting tubes, the laser beam emitted by the system cannot be distributed on the surface of the tumour homogeneously. Therefore, when the site of laser irradiation was changed, we found that different parts of the mass emitted bright upconversion fluorescence, indicating that the distribution of UCNPs in the tumour was not confined to one site but was distributed over multiple sites. However, we also found from the upconversion imaging that the distance between the head of the laser emission tube and tumour on the back was too close, causing the formation of two thick black defects, as shown in (Figure 13A). Although the imaging method had some disadvantages, they were outweighed by the advantages, and this method has potential for application in the early diagnosis and targeted location of lymphoma.

In addition, luminescent nanoparticles were detected in the deeper areas of the subcutaneous mass, and the NIR radiation did not cause obvious damage to the tumour skin following detection. The health of the mice was unaffected, further proving that UCNPs are non-toxic, and these findings were consistent with these of previous reports [38–41].

We believe that the antibody-functionalized UCNPs can also be used for the targeted detection of MCL metastasis. To confirm this idea, we extracted the mouse organs and prepared HE and immunohistochemical sections. From the HE and immunohistochemical images, we can clearly see a slice of tumour metastasis in the liver. During the preparation of the HE slices, various steps were carried out, such as high-temperature baking and haematoxylin, eosin, alcohol, xylene, hydrochloric acid, ammonia and other chemical solvent immersion. However, these steps did not weaken the luminescence of the nanoparticles they still emitted intense UCL upon excitation at 980 nm. As described by Zhou Jing [42] et al, the UCNPs had excellent light stability.

Finally, we used a Ti-S Eclipse inverted fluorescence microscope with a 980 nm near-infrared laser to investigate the position relationship between the nanoparticles and cells in the HE slices. We observed bright UCL in the tumour metastasis but not in the surrounding normal tissues. Therefore, we conclude that the UCNP-antibody conjugates not only can be used to specifically detect primary tumour lesions but are also promising for the detection of tumour metastasis in organs.

From this experiment, we confirmed that UCNP-CD20/CD5 antibody conjugates can be used as biological probes for the early diagnosis of MCL. Upon 980 nm excitation, the MCL cell surface would emit bright double-colour upconversion fluorescence. In addition, the anti-CD20 monoclonal antibody known as rituximab is a chemo-therapeutic drug, which has been widely used in the treatment of NHL derived from B-cells [43–45]. The anti-tumour mechanism of rituximab is mainly dependent on antibody-dependent cell cytotoxicity (ADCC) and complement-dependent cytotoxicity (CDC) [46–47]. The combination of rituximab and CD20 antigen can induce the apoptosis of tumour cells and promote chemotherapy sensitivity. Therefore, using this method, we not only can detect MCL lesions but, in the future, can also administer targeted, precise treatment for MCL lesions and reduce unnecessary damage to normal cells caused by drugs. In conclusion, after modification with the appropriate antibody, UCNPs showd superior performance in early diagnosis and targeted position. In future clinical applications, UCNPs also have potential for use in the precise treatment of cancer.

## MATERIALS AND METHODS

### Materials

NaYF_4_: Er^3+^ and NaYF_4_: Yb^3+^/Tm^3+^ nanoparticles were kindly provided by the electron microscopy room of Mudanjiang Medical University. 2-Morpholinoethanesulfonic acid (MES, 99%), 1-(3-dimethylaminopropyl)-3-ethylcarbodiimide hydrochloride (EDC·HCl, 98%) and N-hydroxysuccinimide (NHS, 98%) were purchased from Shanghai Aladdin Reagent Inc. (Shanghai, China). Hydrogen peroxide solution (H_2_O_2_, 30%) was purchased from Liaoning Quan Rui Reagent Co., Ltd. (Liaoning, China). CD20 (Clone: 2H7), CD20 (Clone: L26) and CD5 (Clone: UCHT2) monoclonal antibodies were purchased from BD Biosciences (San Jose, CA, USA). The Jeko-1 cell line was purchased from the Cell Resource Center of the Shanghai Institute of Life Sciences, Chinese Academy of Sciences (Shanghai, China). The SP50B cell line was kindly provided by Prof. Tadashi Yoshino, Department of Pathology, Okayama University (Japan). SCID Beige SPF-grade mice were purchased from Beijing Weitong Lihua Experimental Animal Technology Co., Ltd. (Beijing, China). RPMI 1640 culture medium and foetal bovine serum (FBS) were purchased from Gibco (USA).

### Preparation of nanoparticles coupled with the CD20/CD5 antibody

#### Conversion of hydrophobic nanoparticles to hydrophilic nanoparticles

Before coupling with antibody, the nanoparticles were converted into hydrophilic nanoparticles. To this end, the C=C double bond of the oleate ligands on the surface of the nanoparticles was oxidized using the strong oxidizer hydrogen peroxide. Next, 0.1 g of hydrophobic NaYF_4_: Yb^3+^/Tm^3+^ nanoparticles was mixed with 8 ml of hydrogen peroxide and stirred for 1 h with magnetic stirring. After collection by centrifugation, the precipitate was washed thoroughly using deionized water in a cell ultrasonic grinding mill and then dried at 70°C for 24 h to obtain hydrophilic NaYF_4_: Yb^3+^/Tm^3+^ nanoparticles.

Similarly, the hydrophilic NaYF_4_: Er^3+^ nanoparticles were also prepared by the above method using NaYF_4_: Er^3+^ nanoparticles as the starting material.

#### Conjugation of oxidized nanoparticles with the CD20/CD5 antibody

Hydrophilic NaYF_4_: Yb^3+^/Tm^3+^ nanoparticles (0.05 g) were added to 10 ml of MES, and the mixture was fully crushed for 5 min using an ultrasonic cell crusher. The power of the ultrasonic cell crusher was set at 600 W. The mixture was subsequently centrifuged for 15 min at 4000 rpm, and the supernatant was discarded. Next, 14 ml of MES, 0.005 g of EDC and 0.015 g of NHS were added to the precipitate, and the mixture was thoroughly stirred for 2 h at room temperature. Following magnetic stirring, the mixture was centrifuged, and the supernatant was discarded. Phosphate-buffered saline (PBS) was added to the precipitate, which was thoroughly washed twice using the ultrasonic cell crusher; the mixture was then centrifuged, and the supernatant was discarded. Next, 5 ml of PBS and 50 μl of CD5 antibody (1:100 dilution) were added to the above precipitate, and the mixture was oscillated for 2 h. The solution with antibody was then stored in the refrigerator at 4°C and was used in subsequent experiments.

Similarly, the NaYF_4_: Er^3+^ nanoparticles coupled with the CD20 monoclonal antibody were also prepared by the above method using the hydrophilic NaYF_4_: Er^3+^ nanoparticles as a starting material.

### Characterization of UCNPs

#### Detection of the upconversion capability for UCNPs

An appropriate amount of UCNP suspension coupled with the corresponding antibody was subjected to performed the upconversion imaging using the NightOWL small-animal imaging system with a 980 nm near-infrared laser. The excitation power values were 0 W, 0.7 W and 1 W respectively.

#### Fourier spectrum analysis of UCNPs

Three types UCNPs, namely, oxidized UCNPs, UCNPs reacted with NHS and EDC and UCNPs reacted with the corresponding antibody, underwent spectrum analysis by FT-IR to verify whether the particles had been successfully bio-functionalized.

#### Detection of cytotoxicity for UCNPs

Two hundred microliters of Jeko-1 cell suspension (number of cells: 6.3×10^5^) was inoculated onto a 24-well culture plate; 12 wells represented the experimental group, and 3 wells represented the control group. We added 800 μl of fresh medium to the above 15 culture wells. The 12 wells from the experimental group were divided into 3 groups, each of which comprised 4 wells. For each group, 20 μl, 40 μl, 80 μl, or 160 μl of UCNP-CD20 antibody conjugate suspension was added to the 4 wells. Finally, the 24-well culture plate was cultured for 24 h, 48 h and 72 h at 37°C and 5% CO_2_. The cells were stained with Trypan blue at different time points, and the viable cells were counted in a blood cell counting plate. The relative cell viability (%) related to the control wells containing cell culture medium without the UCNP-CD20 antibody conjugate suspension was calculated by the following formula:

cell viability (%) = [A]_expt_/[A]_control_×100%, where [A]_expt_ is the number of test samples and [A]_control_ is the number of control samples.

### Cell culture and imaging

The cells were cultured in RPMI1640 medium containing 15% foetal bovine serum (FBS) at 37°C and 5% CO_2_. We used cells in logarithmic growth for experiments. To distribute the cells on the slide homogeneously, 100 μl each of the Jeko-1 or SP50B cell suspension (cell density: 1.5×10^5^/ml) was applied to a cell rejection tablet machine and centrifuged for 5 min at 1000 rpm. The cells were fixed using acetone for 10 min and were washed three times with PBS. Subsequently, 300 μl of a mixture containing 150 μl of the NaYF_4_: Yb^3+^/Tm^3+^ nanoprobe suspension with the CD5 antibody and 150 μl of the NaYF_4_: Er^3+^ nanoprobe suspension with the CD20 antibody was dropped onto the Jeko-1 and SP50B cell region, and the samples were incubated for 2 h at room temperature in the wet box. In the control experiment, 300 μl of a mixture containing 150 μl of the NaYF_4_: Yb^3+^/Tm^3+^ nanoprobe suspension with the CD5 antibody and 150 μl of the NaYF_4_: Er^3+^ nanoprobe suspension without the CD20 antibody, 300 μl of a mixture containing 150 μl of thel NaYF_4_: Yb^3+^/Tm^3+^ nanoprobe suspension without CD5 antibody and 150 μl of the NaYF_4_: Er^3+^ nanoprobe suspension with the CD20 antibody and 300 μl of a mixture without any antibody but containing 150 μl of the NaYF_4_: Yb^3+^/Tm^3+^ nanoprobe suspension and 150 μl of the NaYF_4_: Er^3+^ nanoprobe suspension were dropped on the Jeko-1 and SP50B cell region, and the samples were incubated for 2 h under the same conditions. After incubation, the nanoprobes that did not successfully combine with cells were removed by washing with PBS 3 times. Cell imaging was performed using a Nikon Ti-S Eclipse inverted fluorescence microscope equipped with a 980 nm laser.

### Animal imaging

Six-week-old SCID Beige SPF-grade mice were injected subcutaneously with 0.7 ml of SP50B or Jeko-1 cell/PBS suspension (cell number: 2×10^7^). Approximately 1 month later, the tumour was approximately 0.5 cm in diameter. Next, 500 ul of CD20 antibody-NaYF_4_: Er^3+^ nanoprobe suspension and 500 μl of CD5 antibody-NaYF_4_: Yb^3+^/Tm^3+^ nanoprobe suspension were injected via the tail vein for different mice. Following the intravenous injection of the UCNP-antibody conjugate suspension, the mice were anaesthetized and imaged *in vivo* 12 h, 48 h, 96 h, and 126 h after injection using the NightOWL *in vivo* small-animal imaging system with a 980 nm near-infrared laser whose excitation power and exposure time were 3 W and 10 s, respectively. At the end of the experiment, the animals were euthanized according to the regulations of the management of experimental animals.

### Imaging of organs after embedding with wax

After the mice were euthanized, the liver, spleen, kidney, heart, brain and lungs of the mice were soaked in 80% ethanol. The next day, these organs were embedded in wax and stored in a 4°C refrigerator. After the wax blocks were completely solidified, they were upconversion imaged using the NightOWL small-animal imaging system with a 980 nm laser. The near-infrared laser excitation power was 2 W, and the exposure time was 10 s.

### Imaging of tissue sections after HE staining

After the tissue sections were prepared, they were dyed in accordance with the standard HE staining procedure. HE slices were imaged using the NightOWL small-animal imaging system equipped with a 980 nm laser and Nikon Ti-S Eclipse inverted fluorescence microscope. The near-infrared laser excitation power was 1 W, and the exposure time was 5 s.

## SUPPLEMENTARY MATERIALS FIGURE


