# Supplementary material for: Application of a double-colour upconversion nanofluorescent probe for targeted imaging of mantle cell lymphoma
Source: Oncotarget. 2017 Dec 23;9(24):16758–74. doi: 10.18632/oncotarget.23860 (PMC5908284; doi:10.18632/oncotarget.23860)
Supplement: Supplementary file 1 [file oncotarget-09-16758-s001.pdf]

Application of a double-colour upconversion nanofluorescent probe for targeted imaging of mantle cell lymphoma

SUPPLEMENTARY MATERIALS

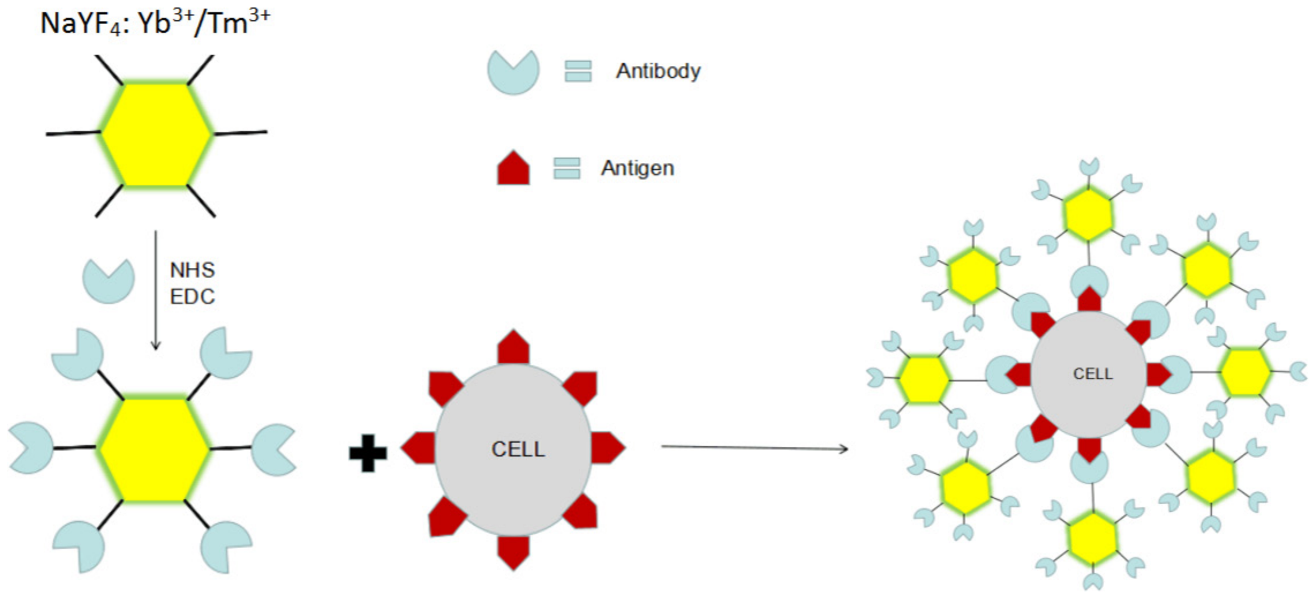

Supplementary Figure 1: Cell immunolabelling.
